# Ischiopubic and odontoid synchondrosis in a boy with progressive pseudorheumatoid chondrodysplasia

**DOI:** 10.1186/1546-0096-5-19

**Published:** 2007-09-27

**Authors:** Ali Al Kaissi, Farid Ben Chehida, Maher Ben Ghachem, Franz Grill, Klaus Klaushofer

**Affiliations:** 1 Ludwig Boltzmann Institute of Osteology, at the Hanusch Hospital of WGKK and AUVA Trauma Centre Meidling, 4 th Medical Department, Hanusch Hospital, Vienna, Austria; 2 Ibn Zohr Institute of Radiology-Imaging Research Department, Tunis, Tunisia; 3 Department of Paediatric Orthopaedic Surgery, Children Hospital, Tunis, Tunisia; 4 Orthopaedic Hospital of Speising, Paediatric Department, Vienna, Austria

## Abstract

**Purpose:**

To present the case of a 14-year-old boy with clinical and radiographic features of pseudorheumatoid chondrodyspalsia with additional, potentially serious, cervical malformations.

**Methods:**

Detailed clinical and radiological examinations were undertaken with emphasis on the usefulness of 3D-CT scanning.

**Results:**

There was synchondrosis between the odontoid and the body of the axis and the cephalad part of the odontoid was detached. Bilateral ischiopubic ossification defects and ischiopubic and odontoid synchondroses were additional abnormalities. 3D-CT scan showed an orthotopic type of os odontoideum associated with an occult axial fracture.

**Conclusion:**

Children who are younger than seven years of age are predisposed to develop odontoid fracture. The latter occur because of the presence of physiological odontoid synchondrosis, but fractures can result from trivial injuries as well as from high-energy trauma. The persistence of an infantile odontoid, with a large pre-adulthood head in children with skeletal dysplasias, is a major risk factor for sudden death or significant morbidity. Comprehensive orthopaedic management must follow early identification of these malformations.

## Introduction

Osteochondrodysplasias are a large heterogeneous group of genetic skeletal dysplasias. Skeletal dysplasias are diagnosed and classified by their clinical phenotype, radiographic features, and their genetic pattern of inheritance [1]

Spranger et al. first described progressive pseudorheumatoid chondrodyspalsia (PPRC) as a progressive connective tissue disease, which combined the radiological features of Scheuermann's disease, with radiographic features of juvenile rheumatoid arthritis [1-3]. The disorder is classified as an autosomal, recessively inherited chondrodyspalsia, with absence of inflammatory parameters. Differentiating progressive pseudorheumatoid chondrodyspalsia (PPRC) from Juvenile rheumatoid arthritis (JRA) is important for Rheumatologists, radiologists and clinicians to further assess and estimate the prognosis and treatment of this generalised bone-cartilage dysplasia syndrome.

In addition our patient had a divided odontoid process, in which the cephalad part was detached from its base, and an os odontoideum was present. This is an abnormality of the upper cervical spine in which the odontoid process is separate from the body of the axis. There was also bilateral, symmetrical, congenital, ischio-pubic synchondrosis which was likely to be congenital in origin, and represents non-fused ends of two separate ossification centres needed for the formation of the ischio-pubic bones [4,5]. To our knowledge, this is the first clinical report of the association of ischiopubic and odontoid synchondrosis in a boy with progressive pseudorheumatoid arthritis.

## Clinical presentation

The patient, a 14-year-old-boy, was referred to the department of pediatric department for clinical assessment because of short stature, progressive joint enlargement, stiffness, pain, rigid and scoliotic back and a progressive waddling gait. The onset was at the age of 6 years and the initial diagnosis at the department of rheumatology was that of juvenile rheumatoid arthritis. However, further investigations revealed no active inflammatory parameters. The patient was born to normal parents who were first-degree relatives. His mother was a 27-year-old-gravida 3, abortus 1, married to a 33-year-old man. Both were healthy, as was an older sister. At birth the patient's length, weight and occipitofrontal circumference (OFC) were around the 10^th ^percentile. His intellectual development was normal.

**Figure 1:**
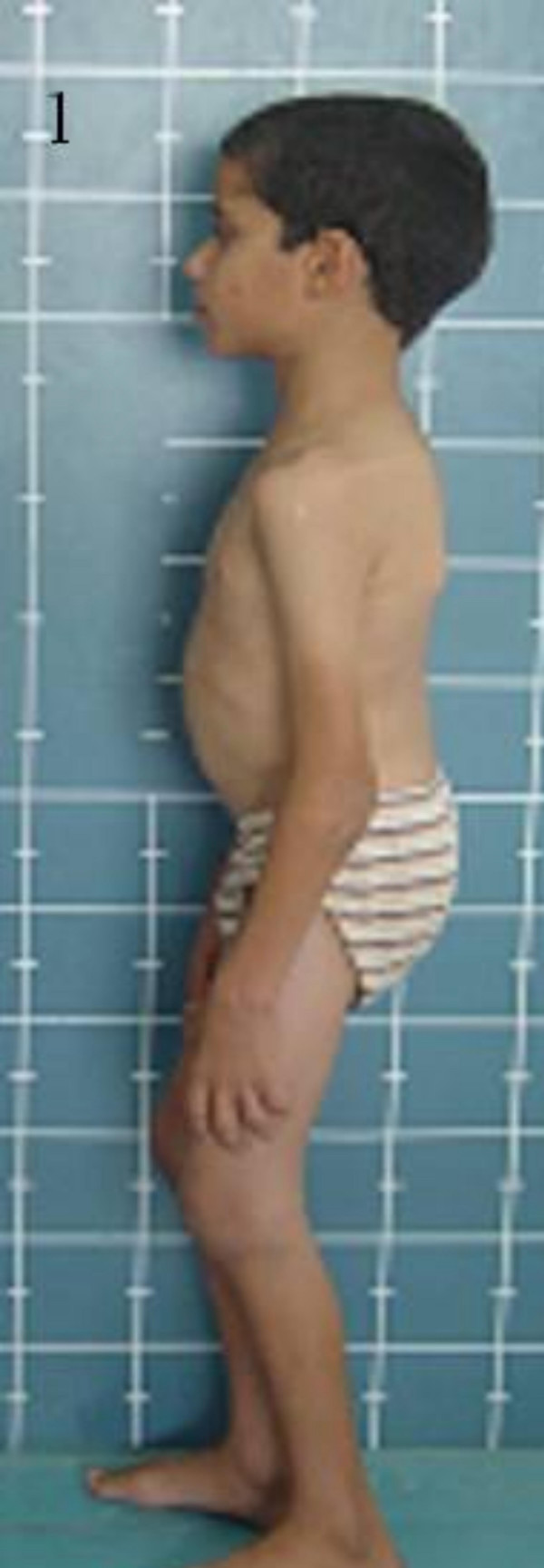
Patient lateral view; note the multiple contractures and the prominent joints.

**Figure 2:**
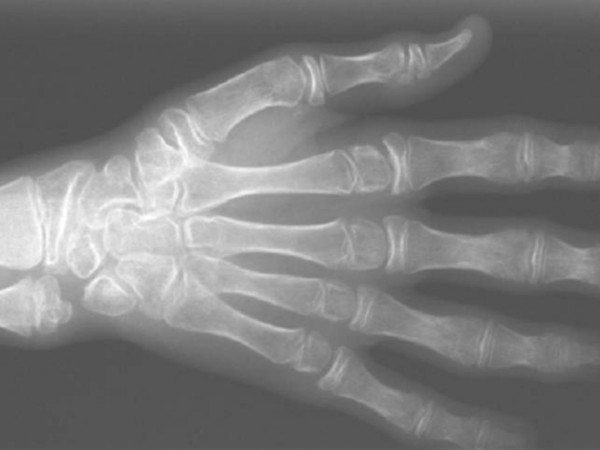
Right hand: Enlargement of the ends of the short tubular bones. Osteopenia of the carpal region with narrowing of the joint spaces.

**Figure 3:**
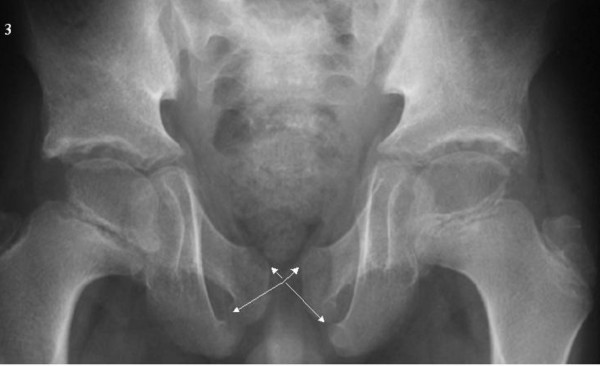
Pelvis: High iliac angles. Broad iliac bodies and femoral necks. Irregular acetabular roofs. Large, and slightly irregular outline capital femoral epiphyses. Bilateral ischiopubic synchondrosis.

**Figure 4:**
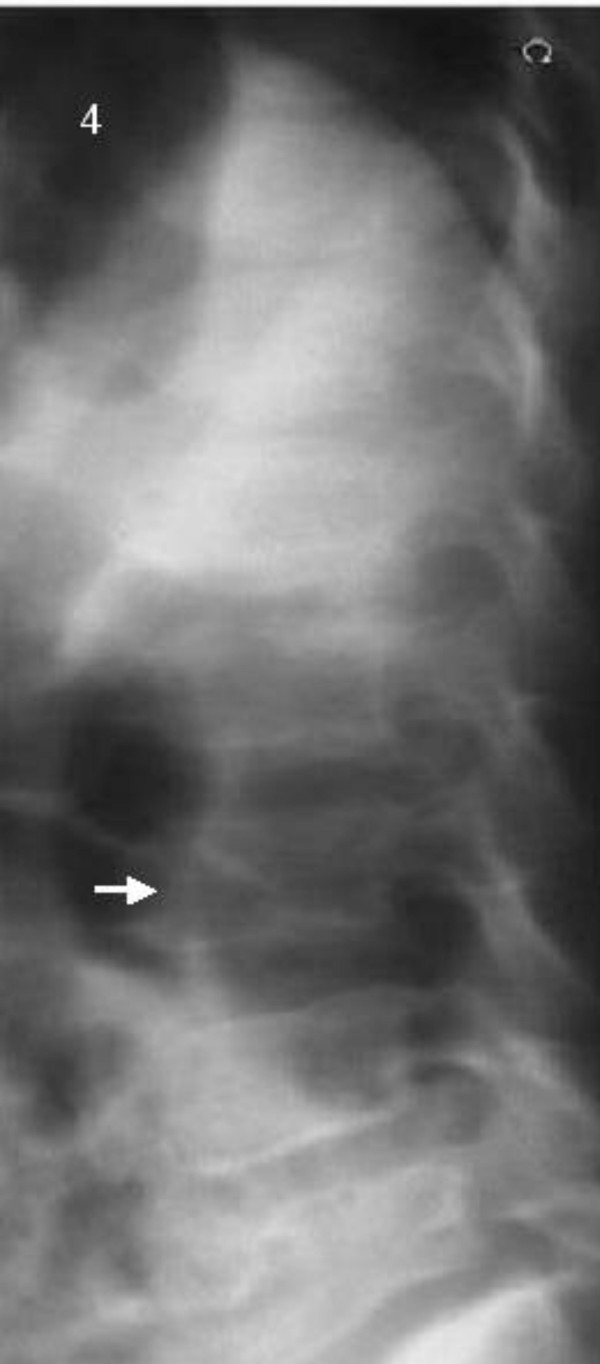
Spinal column: Platyspondyly. Narrowing of the intervertebral disc spaces in the upper lumbar and lower thoracic spine and clefting of the posterior end plates at L2-4 (arrow).

**Figure 5:**
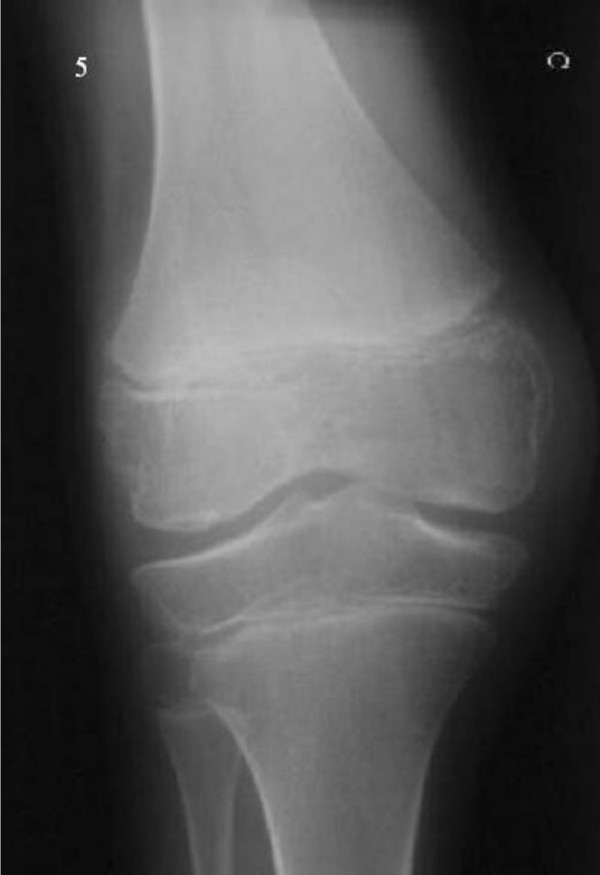
Right knee: Large knee epiphyses. Irregular medial and lateral aspects of the distal femoral epiphyses. Narrowed joint space.

**Figure 6:**
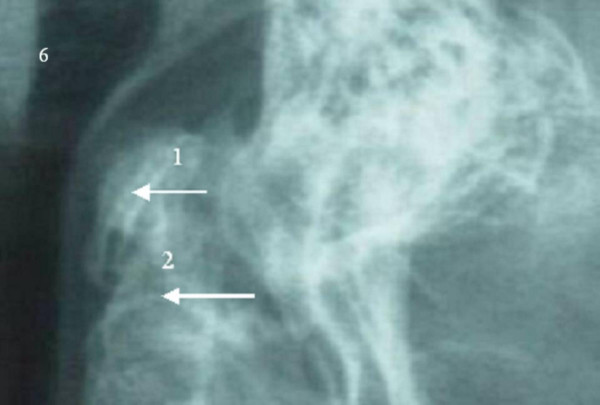
Os odontoideum is seen separated from the odontoid and in relationship to the axis (orthotopic type os, arrow 1). Dynamic x-rays in extension were used to depict the degree of abnormal motion between C1 and C2 (odontoid hypoplasia and axial synchondrosis arrow2). There is anterior instability, with the os odontoideum subluxing forward in relation to the body of C2.

**Figure 7:**
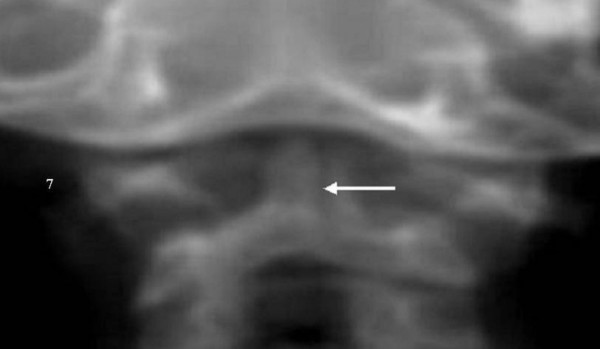
Coronal axial CT scan: Note the abnormal ossification and persistent radiolucencies of the two-paramedian columns. The arrow indicates an occult vertical fracture through the odontoid process and body of the axis.

**Figure 8:**
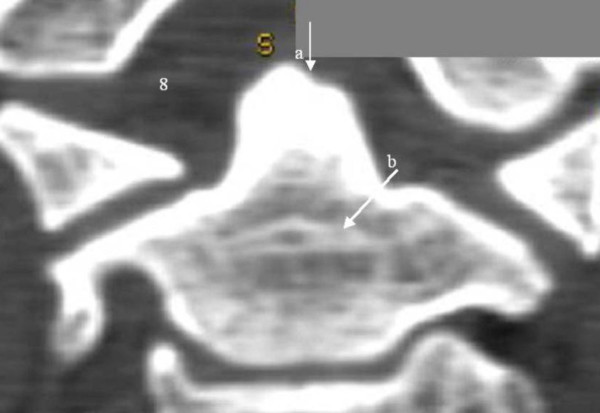
Coronal reconstruction CT scan showed bifid odontoid (arrow1) and subdental synchondrosis (arrow 2).

**Figure 9:**
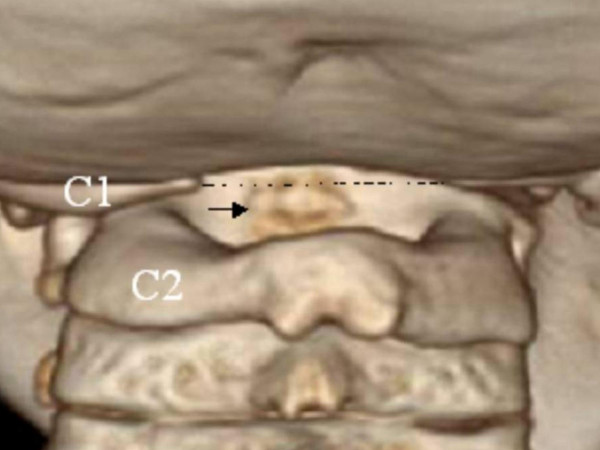
3D reconstruction CT scan showing oval shaped os odontoideum in relationship to the anterior normal arch of the atlas (arrow a); there is marked hypoplasia of the posterior arch of the atlas (arrow b).

When seen at 11 years of age, his height was 120 cm (-4SD), his face was slightly dysmorphic with a prominent forehead, deep-set eyes, long philtrum and thin lips. There was a generalised joint stiffness with multiple contractures (figures 1, 2, 3, 4, 5). All joints were prominent and there was a loss of normal lumbar lordosis. Limitation of neck movements was noticed and it was associated with occasional suboccipital pain (figures 6, 7, 8, 9). There was no history of trauma. Both knees were swollen and felt firm. There was limited flexion and extension of the knees, the wrists and the elbows. Neurological examination was normal as were vision, hearing and mental development. All the routine biochemical tests including erythrocyte sedimentation rate (ESR), rheumatic factors, and antinuclear antibodies were negative.

The child had normal genitalia. All other investigations including an abdominal ultrasound, karyotyping, and metabolic tests, which aimed to test calcium, phosphorus, and vitamin D metabolism, were normal.

Arthroscopy with synovium biopsy gave negative results and juvenile rheumatoid arthritis (JRA) was excluded. The abdominal ultrasound, and Echo-Cardio-Doppler were normal.

Joint pain was treated with nonsteroidal anti-inflammatory medications. Sessions of electrotherapy, and superficial heat (paraffin bath and hot pack) were applied respectively. Special exercises were recommended to increase the muscle strength and to improve the range of joint motion.

On the other hand the odontoid synchondrosis was primarily managed conservatively with regular clinical and imaging surveillance. This will be followed by surgical stabilisation and or/decompression in accordance with the results of the periodic clinical and imaging assessment.

### Radiographic examination

This showed an enlargement of the ends of the short tubular bones of the hands and osteopenia of the carpal region with narrowing of the joint spaces (fig 2).

The pelvis showed high iliac angles, broad iliac bodies and femoral necks, irregular acetabular roofs, and large, slightly irregular, capital femoral epiphyses. Bilateral ischiopubic synchondrosis was present (fig. 3-arrows).

The spinal column showed platyspondyly, narrowing of the intervertebral disc spaces in the upper lumbar and lower thoracic spine, and clefting of the anterior end plates (arrow) in the L2-4 region (fig. 4)

Large knee epiphyses were observed. The distal femoral epiphyses showed irregular medial and lateral aspects and a narrowed joint space (fig. 5).

Os odontoideum was seen separated from the odontoid and in relationship with the axis (orthotopic type os, arrow 1). Dynamic x-rays in extension were used to depict the degree of abnormal motion between C1 and C2 (odontoid hypoplasia and axial synchondrosis, note the anterior angulation of the odontoid process consistent with an odontoid synchondrosis-arrow 2). There was anterior instability, with the os odontoideum subluxing forward in relation to the body of C2 (fig. 6).

Coronal axial CT scan showed an abnormal ossification and persistent radiolucencies in the two-paramedian columns that should have fused in the midline by the 7^th ^foetal month. An occult vertical fracture appeared to run through the odontoid process and body of the axis (fig. 7, arrow).

Coronal reconstruction CT scan showed a bifid odontoid (fig. 8, arrow a) and subdental synchondrosis (fig 8 arrow b)

3D-reconstruction CT scan revealed an oval-shaped os odontoideum incorporated and impacted to the anterior normal arch of the C1 (black arrow), and marked agenesis of the posterior arch of the atlas (dots) C2.

## Discussion

Skeletal dysplasias with early degenerative arthritis or osteoarthritis and are relevant for the differential diagnosis of juvenile idiopathic arthritis and or juvenile rheumatoid arthritis are Stickler syndrome (ophthalmo-arthropathy syndrome), spondyloepiphyseal dysplasia tarda and (PPRC)[1-3,6]. Osteochondrodysplasias are caused by mutations in the genes for structural proteins of connective tissue. El-Shanti et al., [7] and Fisher et al., [8] mapped the gene to 6q22, close to the COL10A1 gene. Mutations could not be found in this gene but it was not totally excluded. Hurvitz et al., [9] demonstrated mutations in the WISP3 gene. This codes for a member of the CCN gene family, which includes cysteine-rich secreted proteins with roles in cell growth and differentiation

Clinically, progressive pseudorheumatoid chondrodyspalsia (PPRC) can be confused with rheumatoid arthritis, but, the main radiological features of (PPRC) are those of spondyloepiphyseal dysplasia with osteoporosis. The phenotypic and radiographic abnormalities in children with PPRC have been extensively described in previous reports [1-3,6,7,10]. Few publications have reported additional abnormalities. Oestreich [11] reported that in patients with PPRC, the os trigonum in the foot was larger than normal and Marek et al. [12] described metatarsal dysplasia in 4 members of a Czech family. Our patient had upper cervical spine abnormalities.

The axis has the most complex and unique development of all vertebrae. There are four ossification centres at birth: one for each neural arch, one for the body, and one for the odontoid process. The odontoid process forms in utero from two separate ossification centres that fuse in the midline by the 7^th ^foetal month. A secondary ossification centre appears at the apex of the odontoid process (os terminale) between 3–6 years of age and fuses by age of 12 years. The body of the axis fuses with the odontoid process by 3–6 years of age. This fusion line (subdental synchondrosis), or the remnant of the cartilaginous synchondrosis, can be seen until age of 11 years and may be confused with a fracture [13].

Lachman [14] reviewed 2100 cases, of well-described skeletal dysplasias, unclassified skeletal dysplasias and syndromes listed in his computerised database. He concluded that subluxations, dislocations, odontoid aplasia or hypoplasia, and cervical kyphosis often occur in patients with skeletal dysplasias. Mild odontoid hypoplasia was the only abnormality noted (PPRC), but no detailed radiographic features were reported and sophisticated techniques were not applied.

Our patient had os odontoideum. Fielding et al. [15] reviewed 35 patients with acquired type os odontoideum from 3 orthopaedic centres, including 14 patients in their first decade of life, 7 in the second decade, and the rest between 20–65 years of age. Nine patients had no history of previous injury. This patients group included two cases of congenital spondyloepiphyseal dysplasia, one case of multiple epiphyseal dysplasia, one case of Down's syndrome, one case of complicated Klippel-Feil syndrome, and four cases of otherwise normal individuals. Interestingly, in 11 patients the os odontoideum was attributed to injury before patients were 4 years old. No patient in this series had features of PPRC.

Currarino [16] reviewed children with diastrophic dysplasia, spondylo-epiphyseal dysplasia congenita, pseudoachondroplasia, Larsen syndrome, and Gorham syndrome. He noted that the odontoid process consisted of two segments (bipartite dens), with an extra ossicle (ossiculum terminale) near the proximal end of odontoid process. He concluded that the appearance of the odontoid process in these patients was anomalous. PPRC did not feature in the group of the patients described.

Ischiopubic ossification defects have been connected to spine abnormalities in a number of remarkable reports [17-19].

The potential risk for both sudden death and significant morbidity as a result of instability of the upper cervical spine in os odontoideum has been extensively illustrated in the literature, highlighting the importance of the early detection of this condition [20-22]. A hypoplastic odontoid is associated with a wide variety of skeletal dysplasias [1,23]. Our patient presented with a detached os odontoideum and the likelihood is that an occult fracture of the axis occurred in conjunction with the remnants of the persistent vestigial cartilaginous synchondrosis. Normal children younger than 7 years of age who present with neck pain or neurological deficits raising concerns about spinal cord injury, an odontoid synchondrosis fracture should be ruled out. In children with skeletal dysplasias however, the impact of trivial injury might be much more drastic, and recommendations for ultimate screening is necessary.

We believe, that the constellation of adverse factors (such as a large pre-adulthood head, and the persistence of cartilaginous axis) predisposed to the detachment of the odontoid process and the development of an occult axis fracture. Finally, we highly recommend the CT scan as a valuable neuroimaging technique for evaluation of axial abnormalities in patients with skeletal dysplasias.
